# MiR-423-5p in brain metastasis: potential role in diagnostics and molecular biology

**DOI:** 10.1038/s41419-018-0955-5

**Published:** 2018-09-17

**Authors:** Guogui Sun, Xiao Ding, Nan Bi, Lihong Wu, Jingbo Wang, WenJue Zhang, Xin Dong, Ning Lv, Yongmei Song, Qimin Zhan, LuHua Wang

**Affiliations:** 1 Department of Radiation Oncology, National Cancer Center/Cancer Hospital, Chinese Academy of Medical Sciences and Peking Union Medical College, Beijing, 100021 China; 2 Department of Radiation Oncology, North China University of Science and Technology Affiliated People’s Hospital, Hebei, 063001 China; 3 Department of Pathology, National Cancer Center/Cancer Hospital, Chinese Academy of Medical Sciences and Peking Union Medical College, Beijing, 100021 China; 4 Department of Radiation Oncology, Shandong Provincial Hospital, Shandong, 250021 China; 5 State Key Laboratory of Molecular Oncology, National Cancer Center/Cancer Hospital, Chinese Academy of Medical Sciences and Peking Union Medical College, Beijing, 100021 China

## Abstract

During the last several years, a growing number of studies have shown that microRNAs (miRNAs) participate in cancer metastasis. Brain metastasis (BM) is a frequent complication of lung adenocarcinoma (LAD), and the incidence of locally advanced LAD with BM can be as high as 30–50%. This study was performed to identify the miRNA expression patterns of LAD with BM and to determine the biological role that miRNAs play in tumorigenesis. To this end, we conducted microarray and quantitative PCR analyses to evaluate BM-related miRNAs independently validated from a total of 155 patients with LAD. A series of in vivo and in vitro assays were also conducted to verify the impact of miRNAs on BM. We found significantly increased expression of miR-423-5p, and BM was predicted in non-small cell lung cancer when compared to LAD without BM. We next examined the function of miR-423-5p and discovered that it significantly promoted colony formation, cell motility, migration, and invasion in vitro. We computationally and experimentally confirmed that metastasis suppressor 1 (*MTSS1*) was a direct miR-423-5p target. Through a combination of image, histological, and molecular analyses, we found that miR-423-5p overexpression significantly increased tumor burden, local invasion, and distant BM. The level of MTSS1 expression was inversely correlated with miR-423-5p upregulation in the LAD specimens and was associated with survival of patients with BM. MiR-423-5p promoted BM in LAD and inhibited MTSS1 expression. Together, these results show that MiR-423-5p has the potential to be a marker of BM and/or a therapeutic target in LAD.

## Introduction

Lung cancer is the leading cause of cancer-related death worldwide, and as such, is a serious threat to human health and well-being^1^. A total of 80% of lung cancer cases are attributable to non-small cell lung cancer (NSCLC), which includes two major histologic subtypes: lung adenocarcinoma (LAD) and squamous cell carcinoma^2,3^. Brain metastasis (BM) from lung cancer is one of the principal causes of morbidity and mortality because even tiny satellite lesions can cause impairment. About 40% of LAD patients end up with BM at some point during their illness^3^. The cumulative incidence of BM in NSCLC is 16–35% and is linked to a poor prognosis^4,5^. In patients with BM in NSCLC, the overall survival ranges from 3.0 to 14.8 months, when stratified by lung-specific graded prognostic assessment scores^6^. BM also induces substantial cognitive, neurological, and emotional issues^7^, and ultimately, poor survival^8^. There are currently no common measures that are used to reduce the risk of BM in NSCLC. Nevertheless, upon diagnosis, BM is identified in about 10% of all lung cancer patients; in several retrospective series, BM was documented in 50% of patients^9,10^. Despite progress in the creation of molecularly targeted therapies for primary lung tumors, the majority of lung cancer deaths occur due to the substantial growth of metastases that are immune to traditional therapies.

Thus, identifying biomarkers that allow the accurate detection of early changes in tumors and the molecular characteristics of BM is the principal goal for the management of LAD. Currently, prophylactic cranial irradiation (PCI) is suggested for every patient with small cell lung cancer (but not with NSCLC) in the early stages of the disease or for patients with stable disease following their first systemic treatment^11^. Nevertheless, improving the selection of the patients who should receive PCI will save patients from unnecessary treatments, as they are impossible to develop BM from PCI-related side effects. In locally advanced stage III LAD, a clinical trial to establish the advantage of PCI accumulated data slowly, was terminated early, and did not have the statistical power to achieve the primary goal of improvement in survival^12^. To the best of our knowledge, no commonly used procedures exist to lower BM risk in LAD patients. Therefore, identifying early tumor alterations linked to BM via pertinent biomarkers could be integrated in clinical decisions as prognostic indicators to personalize treatments and follow-up plans. Molecular biomarkers may be advantageous in the stratification of these patients, but it can be challenging and limiting to acquire appropriate, substantial amounts of tumor tissue in a systematic manner for genomic profiling. It was recently shown that microRNAs (miRNAs), endogenous small non-coding RNAs, play a pivotal role in modulating numerous cellular activities^13–15^. MiRNAs are highly conserved among species, and a growing number of studies have demonstrated their involvement in cancer metastasis^16^. Examining the miRNA profile of various tumors has increased in popularity over the last 10 years and constitutes a breakthrough in tumor classification that can affect cancer diagnosis, prognosis, and treatment selection^17^. The brain microenvironment regulates metastatic tumor growth, but the exact genetic occurrences that encourage metastasis in the brain niche have been a subject of debate. When these occurrences are better understood, disease-based targets and successful techniques to treat BM will be developed.

Thus, in this study, we focused on current findings about the regulatory roles of miRNAs in LAD cell activities that impact metastasis and discuss the utilization of miRNAs as biomarkers and in miRNA-based therapeutics.

## Results

### Differentially expressed miRNAs were detected in primary specimens from LAD patients with or without BM by expression profiling

**Fig. 1:**
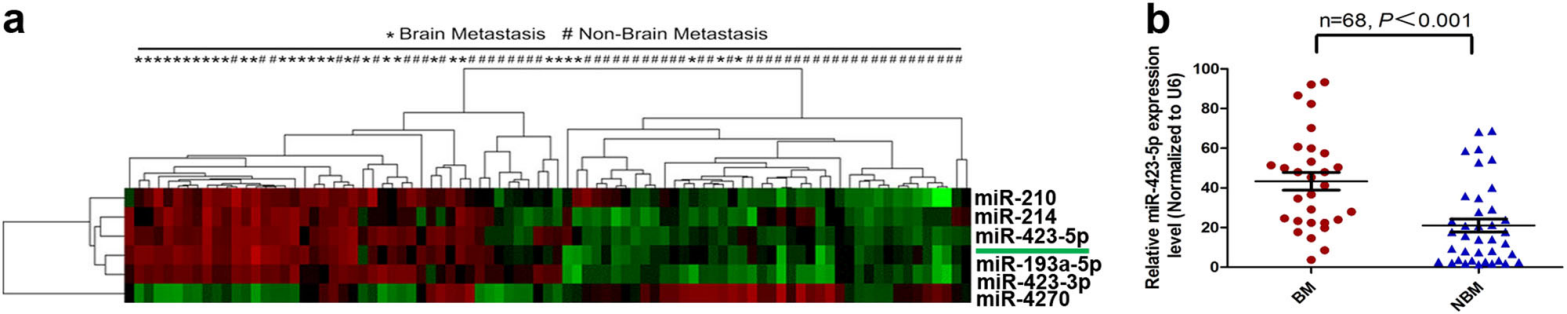
**a** Unsupervised clustering of expression profiling of miRNA of 32 lung LAD cases with BM and 55 BM-free cases (training group). **b** Quantitation of miR-423-5p was performed by qPCR in 30 lung adenocarcinoma cases with BM and 38 BM-free cases (test group)

To detect miRNAs linked with BM in LAD, we compared the miRNA expression profiles in formalin-fixed, paraffin-embedded (FFPE) specimens of 32 LAD patients with BM with 55 patients without BM (Table 1). The normalization of fluorescence signals was conducted using the median center tool for genes in Cluster 3.0 and evaluated using the significance analysis of microarrays, with a false discovery rate threshold set to 0, fold-change established at ≥2 or ≤0.5-fold change, and *P* value < 0.05. A total of six miRNAs were differentially expressed when comparing LAD tissues with and without BM (Fig. 1a). Among these molecules, five miRNAs, miR-214, miR-210, miR-423-5p, miR-193-5p, and miR-423-3p, were significantly upregulated in the BM group, while one miRNA, miR-4270, was downregulated. These evaluations suggested that classification with as few as six miRNA markers might effectively differentiate BM from LAD tissues in Chinese patients. Then we sorted the six differentially expressed miRNAs according to statistical value between LAD tissue with and without BM, and the highest distinguishing values of miR-423-5p were identified. To perform additional analyses, all of the samples were split into high and low expression groups by miRNA microarray analysis, based on the median fluorescence signal values of the six miRNAs. Single Cox regression analysis showed that the most obvious risk ratio (RR) of miR-423-5p was associated with BM in LAD (*RR* = 10.883, Table 2); therefore, we selected this miRNA for further investigation.

**Table 1:** Comparison of clinical characteristics of LAD patients with and without BM

Characteristics	Training group			Test group		
	BM (*n* = 32)	NBM (*n* = 55)	*P*	BM (*n* = 30)	NBM (*n* = 38)	*P*
Gender						
Male	17 (53.1%)	33 (60%)	0.532	17 (56.7%)	25 (65.8%)	0.442
Female	15 (46.9%)	22 (40%)		13 (43.3%)	13 (34.2%)	
Age						
≤60	20 (62.5%)	27 (49.1%)	0.226	13 (43.3%)	16 (42.1%)	0.919
>60	12 (37.5%)	28 (50.9%)		17 (56.7%)	22 (57.9%)	
Tumor stage						
T1 + T2	27 (84.4%)	47 (85.5%)	0.892	28 (93.3%)	34 (89.5%)	0.577
T3	5 (15.6%)	8 (14.5%)		2 (6.7%)	4 (9.5%)	
Histologic grade						
Well/moderate	22 (68.8%)	34 (61.8%)	0.515	9 (30.0%)	13 (34.2%)	0.712
Poor/NS	10 (31.2%)	21 (38.2%)		21 (70.0%)	25 (65.8%)	
Lymph node ratio						
≤1/3	10 (31.2%)	26 (47.3%)	0.143	20 (66.7%)	23 (60.5%)	0.602
>1/3	22 (68.8%	29 (52.7%)		10 (33.3%)	15 (39.5%)	

*BM* brain metastasis, *NBM* no brain metastasis, *NS* not stated

**Table 2:** Multivariate Cox regression analysis of BM with LAD patients according to the expression status of six microRNAs

MicroRNAs	B	*RR*	95%CI	Wald *χ*^2^	*P*
miR- 423-5p	2.387	10.883	3.737-31.699	19.115	0.000
miR- 214	2.353	10.519	3.987-27.754	22.597	0.000
miR-210	2.324	10.214	3.573-29.199	18.800	0.000
miR- 193a-5p	2.302	9.992	3.424-29.160	17.741	0.000
miR- 423-3p	1.829	6.227	2.392-16.214	14.033	0.000
miR-4270	-1.415	0.243	0.100-0.591	9.738	0.000

*RR* relative risk, *B* regression coefficients

### MiR-423-5p was upregulated and predicted BM in LAD patients

To determine if miR-423-5p could be a biomarker for BM in LAD patients, we analyzed the relationship between miR-423-5p expression level and BM in FFPE specimens of 32 LAD patients with BM versus 55 patients without BM. Increased miR-423-5p was significantly correlated with numbers of BM (Table 3). No correlation was observed between miR-423-5p expression levels and age, gender, T stage, histologic grade, or lymph node ratio (Table 3). Kaplan–Meier analysis determined that high levels of miR-423-5p expression were linked to poor BM survival in LAD patients in the training group (Table 4). To validate whether miR-423-5p was increased in LAD tissues with BM, quantitative PCR (qPCR) was used to examine mature miR-423-5p levels in the FFPE specimens of 30 LAD patients with BM and 38 patients without BM (Table 1). MiR-423-5p levels in 30 LAD tissues with BM were markedly higher than those in 38 LAD tissues without BM (Fig. 1b, Table 3). The miR-423-5p expression level was not correlated with gender, T stage, histologic grade, or lymph node ratio (Table 3), but was correlated with age (Table 3). Kaplan–Meier survival analysis confirmed that high miR-423-5p expression was associated with the significantly decreased survival of patients in the test group (Table 4).

**Table 3:** Correlation between miR-423-5p expression and clinicopathological parameters of LAD patients with and without BM

Characteristics	miR-423-5p expression levels of training group			miR-423-5p expression levels of test group		
	Low (*n* = 44)	High (*n* = 43)	*P*	Low (*n* = 34)	High (*n* = 34)	*P*
Gender						
Male	24 (54.5%)	26 (60.5%)	0.577	23 (67.6%)	19 (55.9%)	0.318
Female	20 (45.5%)	17 (39.5%)		11 (33.4%)	15 (44.1%)	
Age						
≤60	20 (45.5%)	27 (62.8%)	0.105	10 (29.4%)	19 (55.9%)	0.027
>60	24 (54.5%)	16 (37.2%)		24 (70.6%)	15 (44.1%)	
Tumor stage						
T1 + T2	40 (90.9%)	34 (79.1%)	0.121	31 (91.2%)	31 (91.2%)	1.000
T3	4 (9.1%)	9 (20.9%)		3 (8.8%)	3 (8.8%)	
Histologic grade						
Well/moderate	29 (65.9%)	27 (62.8%)	0.761	22 (64.7%)	24 (70.6%)	0.604
Poor/NS	15 (34.1%)	16 (37.2%)		12 (35.3%)	10 (29.4%)	
Lymph node ratio						
≤1/3	20 (45.5%)	16 (37.2%)	0.435	22 (64.7%)	21 (61.8%)	0.801
>1/3	24 (54.5%)	27 (62.8%)		12 (35.3%)	13 (39.2%)	
Brain metastasis						
BM	4 (9.1%)	28 (65.1%)	0.000	8 (23.5%)	22 (64.7%)	0.001
NBM	40 (90.9%)	15 (34.9%)		26 (76.5%)	12 (35.3%)	

*BM* brain metastasis, *NBM* non-brain metastasis, *NS* not stated

**Table 4:** Multivariate cox regression analysis of BM with LAD patients

Variable	Training group (*n* = 87)			Test group (*n* = 68)		
	95%CI	*RR*	*P*	95%CI	*RR*	*P*
Sex (male vs. female)	0.479–2.266	1.024	0.917	0.486–2.413	1.087	0.839
Age (≤60 vs. >60 years)	0.245-1.169	0.535	0.117	0.520-2.805	1.208	0.660
Tumor stages (T1 + T2 vs.T3)	0.262–2.300	0.776	0.648	0.202-3.968	0.896	0.885
Histologic grade (Well/moderate vs. Poor/NS)	0.565–2.749	1.246	0.585	0.368–2.199	0.900	0.817
Lymph node ratio (≤1/3 vs. >1/3)	1.417–8.242	3.417	0.006	0.445–2.256	1.002	0.997
miR-423-5p expression levels	3.772–33.660	11.268	0.000	1.387–7.546	3.236	0.007

*RR* relative risk

### MiR-423-5p overexpression promoted colony formation, cell motility, migration, and invasion of LAD cells

**Fig. 2:**
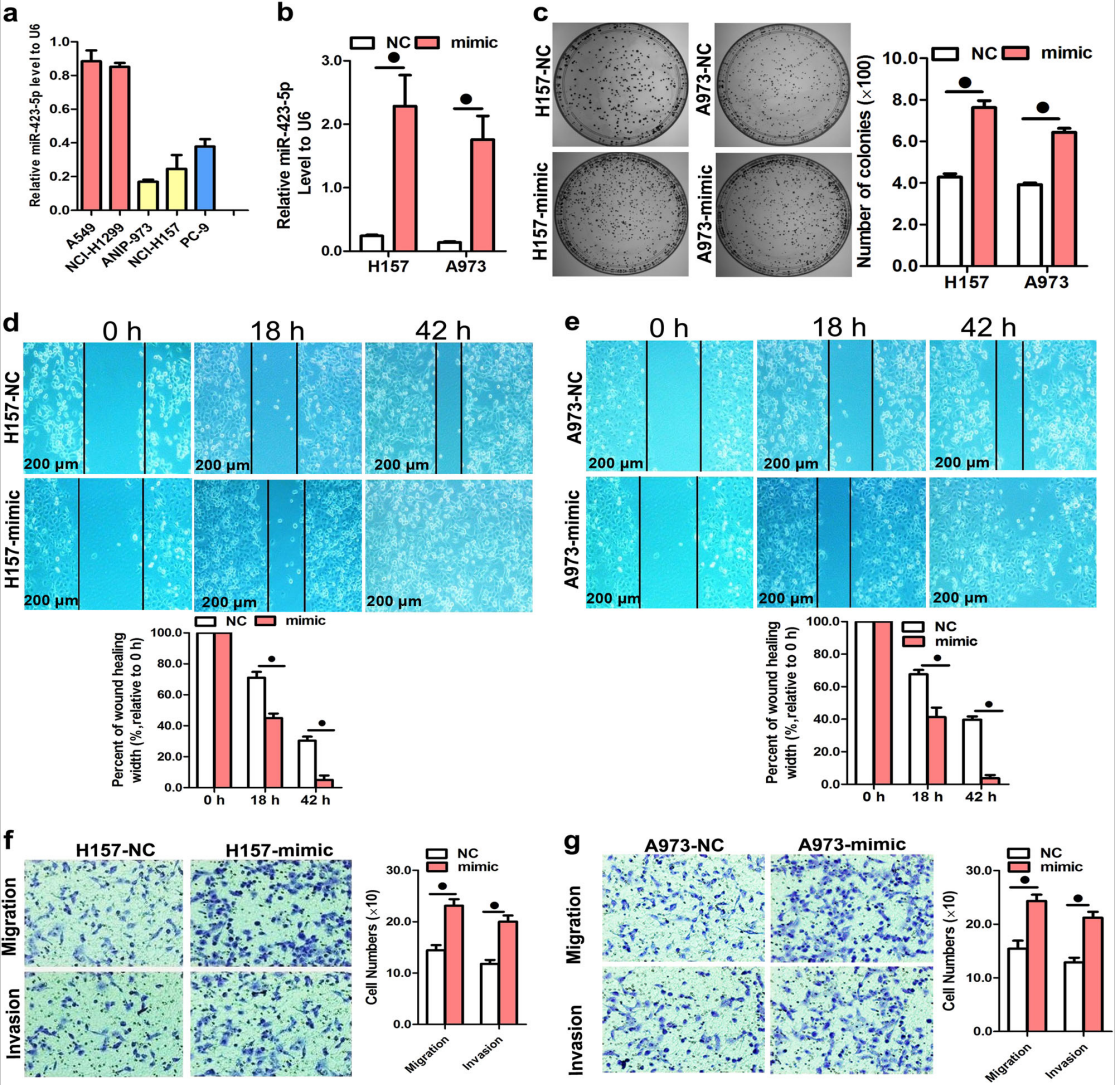
MiR-423-5p overexpression promoted colony formation, cell motility, migration, and invasion. **a** Level of miR-423-5p RNA in five NSCLC cell lines. **b** Quantitation of miR-423-5p levels after the transfection of H157 and A973 cell lines with miR423-5p mimic. **c** Representative images and quantitative results of colony formation were obtained after the transfection of miR-423-5p mimic in H157 and A973 cell lines. **d**, **e** MiR-423-5p overexpression accelerated cell motility, which was measured by the wound-healing assay in the H157 and A973 cell lines. **f**, **g** Representative images and quantitative results of transwell assays were obtained after the transfection of H157 and A973 cell lines with miR-423-5p mimic. Data are the mean ± SD from three experiments. ^●^*P* < 0.05

**Fig. 3:**
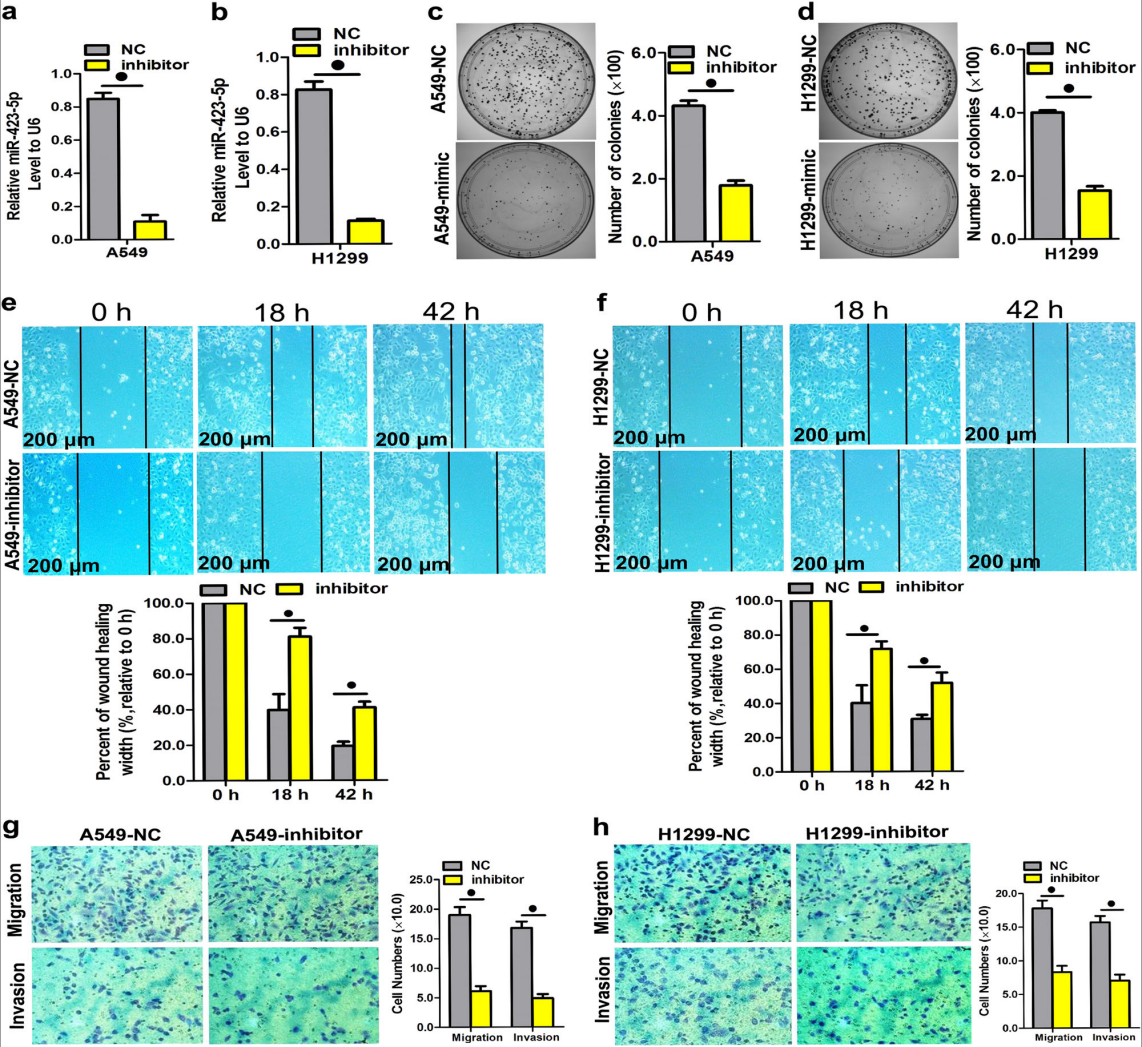
When miR-423-5p expression was suppressed, colony formation was significantly inhibited as well as cell motility, migration, and invasion in A549 and H1299 cells. **a**, **b** Quantitation of miR-423-5p levels after the transfection of A549 and H1299 cell lines with miR-423-5p inhibitor. **c**, **d** Representative images and colony formation quantitation were obtained after the transfection of A549 and H1299 cell lines with miR-423-5p inhibitor. **e**, **f** When expression of miR-423-5p was decreased, cell motility was inhibited, as measured in both the A549 and H1299 cell lines by the wound-healing assay. **g**, **h** Representative images and transwell assay results were obtained after the transfection of A549 and H1299 cell lines with miR-423-5p inhibitor. Data are presented as the mean ± SD of three experiments. ^●^*P* < 0.05

To further investigate the role of miR-423-5p in the regulation of LAD cell colony formation, motility, migration, and invasion, the exogenous expression of miR-423-5p was achieved by transfecting miR-423-5p mimics into H157 and A973 lung cancer cells, in which endogenous miR-423-5p expression was relatively low (Fig. 2a). Transfection efficiency was verified by a significant increase of miR-423-5p expression in H157 and A973 cells, as determined by qPCR (Fig. 2b). We found that the exogenous high expression of miR-423-5p significantly promoted the colony formation of H157 and A973 cells (Fig. 2c). Moreover, the overexpression of miR-423-5p significantly accelerated the rate of wound gap closure in both H157 and A973 cells in the wound healing assay (Fig. 2d). The transwell assay further demonstrated that miR-423-5p overexpression increased the migration and invasion of both H157 and A973 cells (Figs. 2f, g). We also examined the effect of miR-423-5p inhibitors on LAD cell colony formation, motility, migration, and invasion. The A549 and H1299 cells were chosen for the follow-up experiments because of the high miR-423-5p expression levels in these cells (Fig. 2a). The colony formation assay showed that the growth of A549 and H1299 cells was decreased by the transfection of miR-423-5p inhibitors (Figs. 3a–d). Comparably, in wound healing assays, the mobility of A549 and H1299 cells was significantly decreased following transfection with miR-423-5p inhibitors (Fig. 3e). We further investigated whether miR-423-5p could inhibit the migration and invasion of LAD cells, and found that the transfection of miR-423-5p inhibitors significantly repressed A549 and H1299 cell migration and invasion (Figs. 3g, h).

### MiR-423-5p directly targeted MTSS1 to promote proliferation and metastasis

**Fig. 4:**
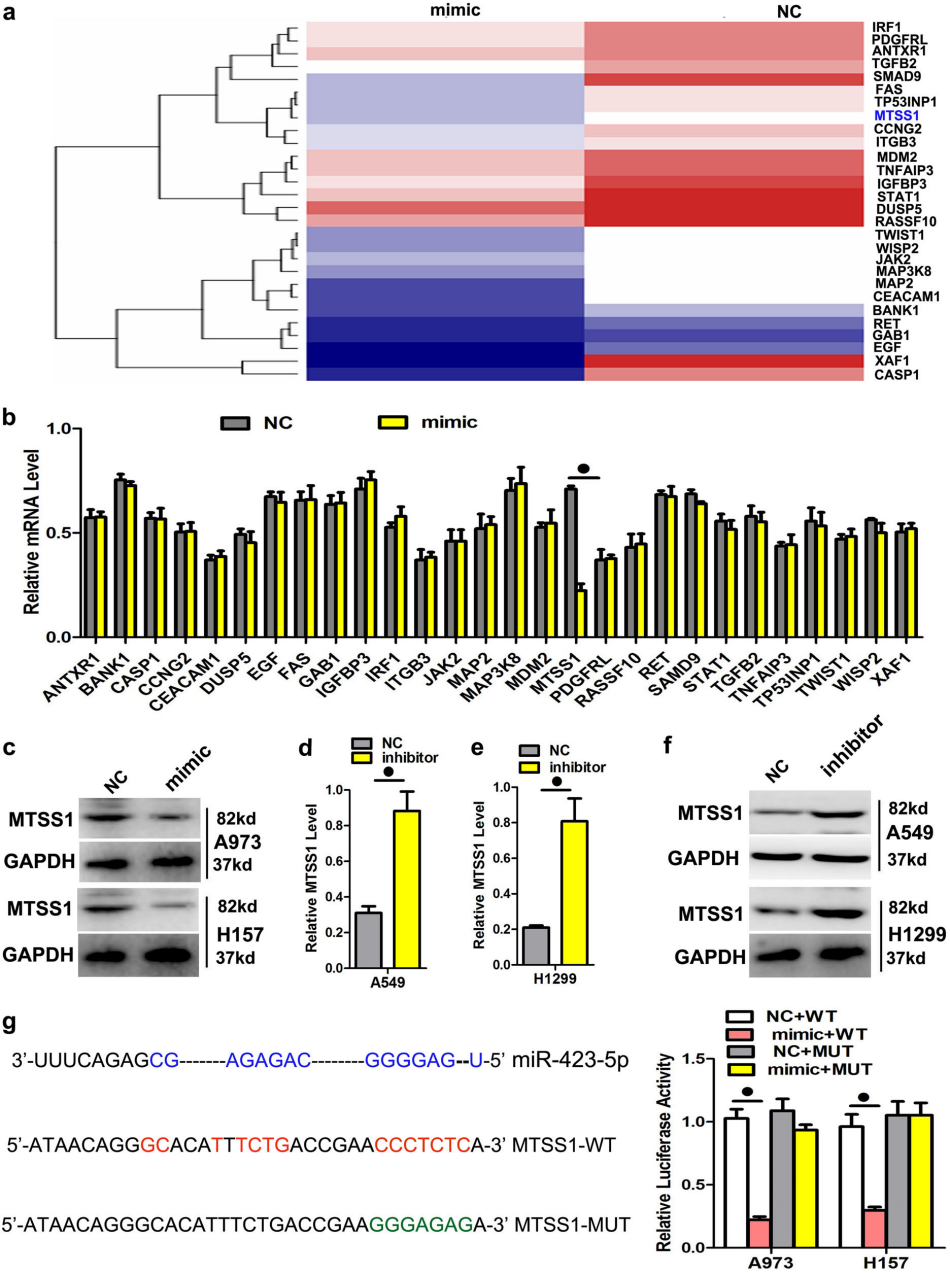
MiR-423-5p directly targets the MTSS1 gene. **a**, **b** MTSS1 was identified as a putative regulatory target of miR-423-5p by considering the downregulated genes using global gene expression analysis and qPCR. **c**, **d** MTSS1 protein and mRNA expression was measured by Western blotting after transfection of H157 and A973 cell lines with the miR-423-5p mimic. GAPDH was the loading control. **e**, **f** qPCR and Western blotting were used to measure MTSS1 mRNA and protein expression levels, respectively, in A549 and H1299 cell lines after transfection with miR-423-5p inhibitors. GAPDH was used as a loading control. g Dual-luciferase reporter assay. Normalization of relative luciferase activity to Renilla luciferase activity assay was performed after co-transfection of H157 and A973 cell lines with the miR-423-5p mimic and pmiR-RB-REPORT™ construct containing wild-type or mutant MTSS1 3‵-UTR region. Data are the mean ± SD of three experiments. ^●^*P* < 0.05

**Fig. 5:**
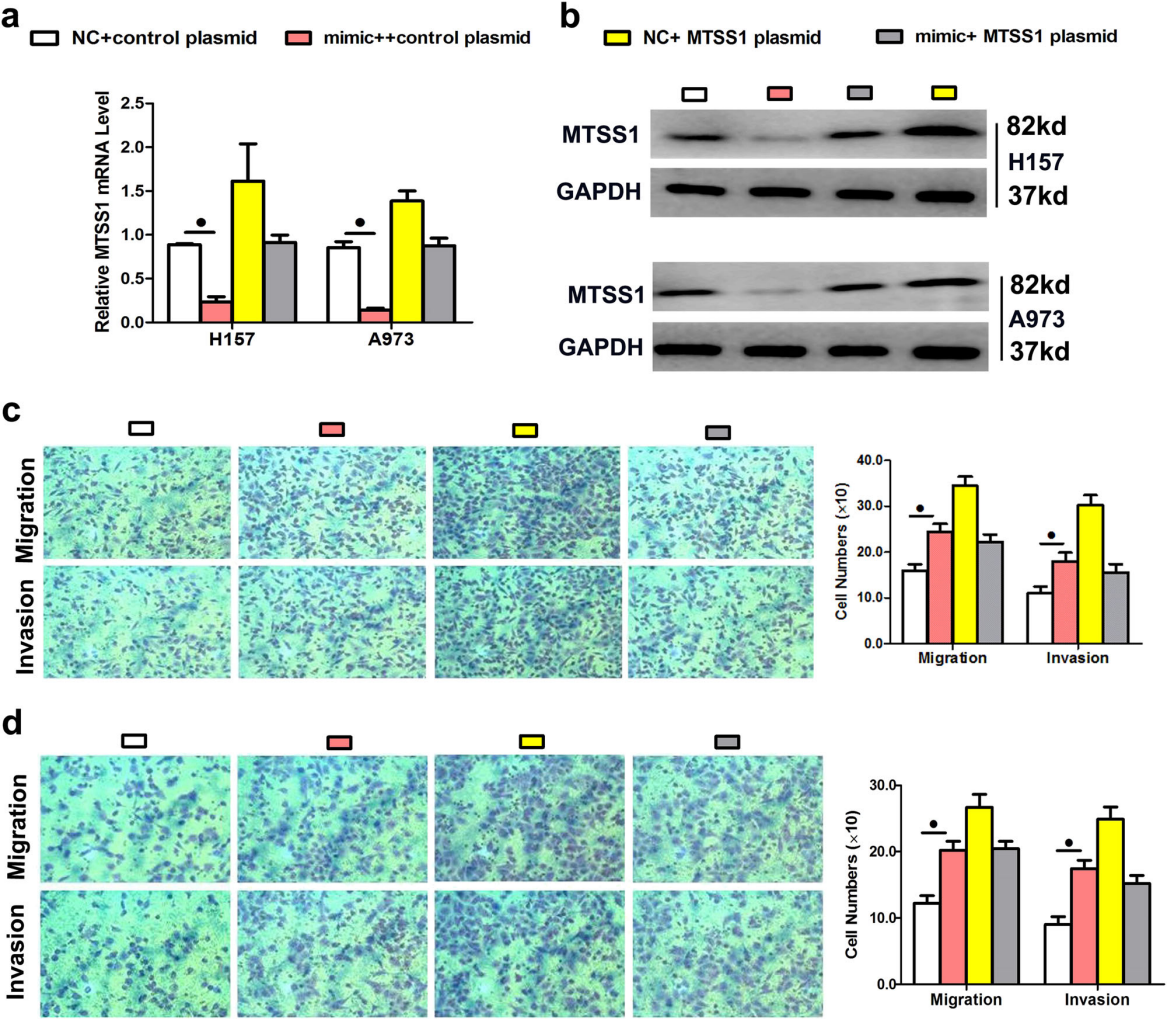
A rescue assay was performed to confirm that MTSS1 was the functional target of miR-423-5p. **a**, **b** MTSS1 RNA and protein levels in A973 and H157 cell lines after co-transfection with the miR-423-5p mimic and pEGFP-C1 plasmid containing the MTSS1 CDS sequence. **c**, **d** Transwell assay after co-transfection of cells with miR-423-5p mimic and MTSS1 plasmid. Data are the mean ± SD of three experiments. ^●^*P* < 0.05

To obtain knowledge about the genes downstream of miR-423-5p that regulate its biological functions, we performed global gene expression analysis using the GeneChip® Affymetrix 3’IVT. A total of 1439 probes were expressed in H157 cells: 303 were upregulated genes and 733 were downregulated genes (fold-change ≥ 1.5, *P* < 0.05) (Fig. 4a). We chose 28 genes and evaluated their mRNA expression levels by qPCR to verify the array outcomes (Fig. 4b). Of these genes, metastasis tumor suppressor-1 (MTSS1) was of special interest because this gene had the highest total context score, and its role in epithelial-mesenchymal transition has been greatly documented in lung cancer. To determine whether miR-423-5p inhibited MTSS1 expression, the miR-423-5p mimic was transiently transfected into H157 and A973 cells, and qPCR and Western blotting were used to measure endogenous MTSS1 expression. At 48 h following mimic transfection, miR-423-5p expression significantly lowered MTSS1 protein levels in every cell line evaluated (Figs. 4c, d). In addition, we transfected LAD cells with miR-423-5p inhibitors to verify the outcomes of mimic transfection. As anticipated, the downregulation of miR-423-5p utilizing inhibitors increased MTSS1 mRNA and protein levels in the A549 and H1299 cells (Figs. 4e, f). Finally, to determine if miR-423-5p downregulated MTSS1 via the putative 3‵-untranslated region (UTR) miR-423-5p target sequences of MTSS1, the sequence was cloned into a luciferase reporter plasmid, various mutants were produced, and the luciferase activities of various constructs in the H157 and A973 cells were measured. Although the luciferase activity of the reporter harboring the wild-type sequence was significantly reduced by miR-423-5p expression, mutation of the potential miR-423-5p target sequences eliminated the effect of miR-423-5p on luciferase activity (Fig. 4g), suggesting that both 3’-UTR miR-423-5p target sequences of MTSS1 are involved in miR-423-5p-mediated regulation. A rescue experiment was conducted to verify that MTSS1 was the functional target of miR-423-5p in the H157 and A973 cells, as MTSS1 mRNA and protein (endogenous) levels in both cell lines were eliminated by mimic transfection and rescued by the transfection of both pEGFP-N1-MTSS1 constructs, respectively (Figs. 5a, b). These outcomes demonstrated that the migration and invasion promoted by mimic transfection were reversed by the transfection of the two expression constructs (Figs. 5c, d).

### MiR-423-5p escalated tumor growth and metastasis in vivo

**Fig. 6:**
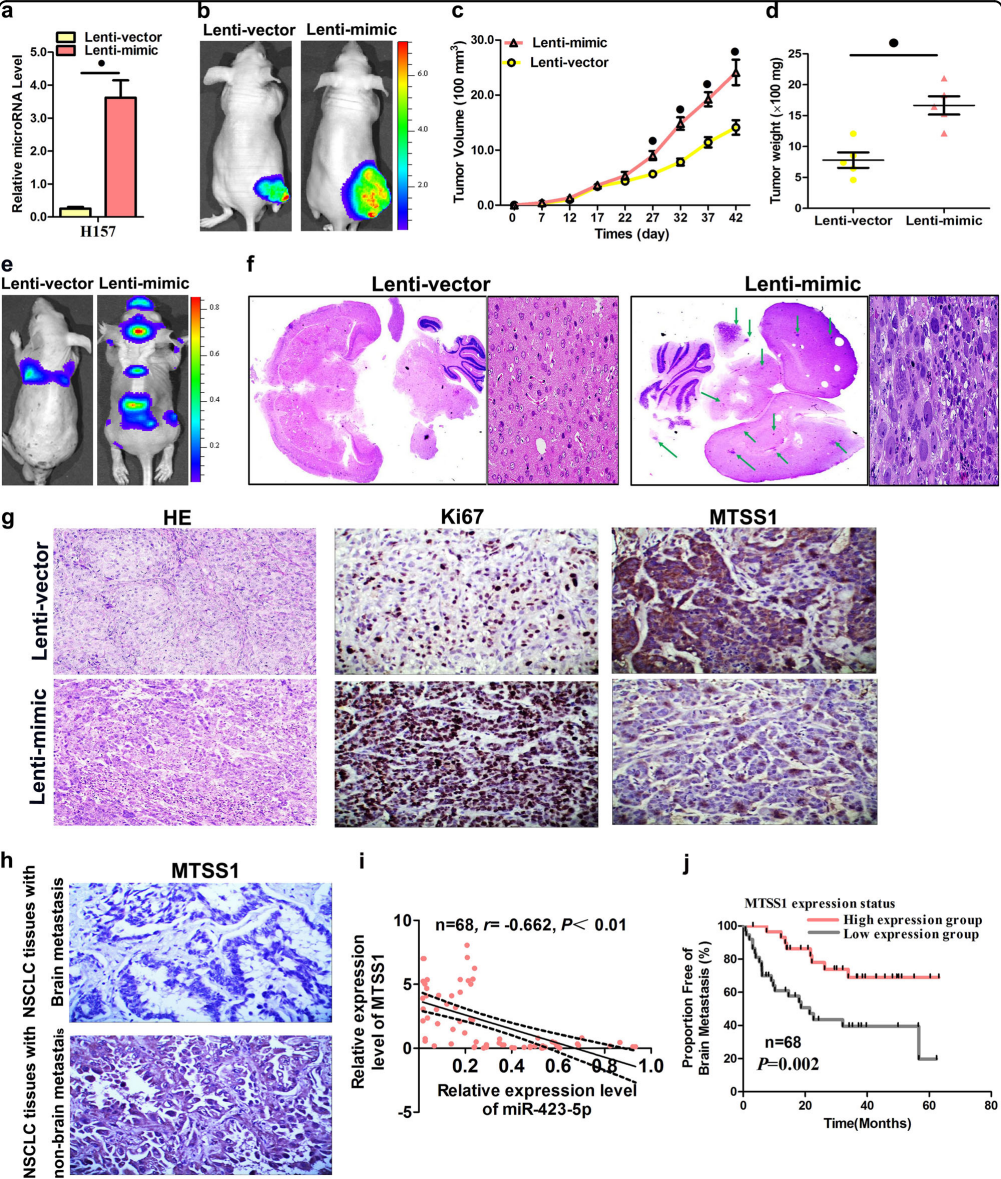
Tumor growth and metastasis was promoted by miR-423-5p overexpression in vivo. **a** Levels of miR-423-5p in stable miR-423-5p-overexpressing (Lenti-mimic) and control (Lenti-vector) H157 cells. **b**–**d** H157 cells stably overexpressing miR-423-5p were injected subcutaneously into nude mice to produce solid tumors, and representative images of tumor volumes and weights were analyzed via in vivo luciferase imaging on the endpoint day (*n* = 5 for each group). **e**, **f** The numbers of metastatic nodules were observed and then quantified in the brains of mice treated with H157 cells that stably overexpressed miR-423-5p or control vector cells by vein injection. **g** Ki67 immunohistochemical staining, and MTSS1 in tumor tissues taken from nude mice overexpressing miR-423-5p compared with the control group. **h** MTSS1 protein expression measured by immunohistochemical staining in LAD samples. **i** MTSS1 expression and association with the survival of LAD patients with BM. **j** Spearman correlation analysis of the negative correlation between MTSS1 and miR-423-5p expression. Data are the mean ± SD of three experiments. ^●^*P* < 0.05

Because the proliferation, migration, and invasion of LAD cells were induced by miR-423-5p in vitro, we evaluated whether miR-423-5p could impact tumorigenicity in vivo. The H157 cells that stably expressed miR-423-5p and the negative control vector were subcutaneously injected into the nude mice. The successful overexpression of miR-423-5p in the H157 cells after lentiviral infection was confirmed by qPCR (Fig. 6a). Starting on day 7 after implantation, the tumor lengths and widths were documented every 5 days for a total of eight measurements. The mice were sacrificed 6 weeks following tumor cell implantation. The tumor growth curve detailed a significant escalation in the miR-423-5p overexpression group in contrast to the control group (Figs. 6b, c). Then the tumors were anatomized, and the exact sizes and weights were examined. In contrast to the control group, the mean volume and mass of the tumors in the miR-423-5p overexpression group were significantly bigger and heavier (Fig. 6d). Approximately 1 × 10^6^ luciferase-labeled cells were intravenously injected into the tail veins of mice for 6 weeks. Luciferase activity was used to evaluate tumor burden in the lung and brain. As expected, miR-423-5p overexpression significantly increased the brain metastasis lesions through tail vein injection (Figs. 6e, f). Together, the results showed that miR-423-5p is important for LAD growth and metastasis in vivo, particularly in BM. Furthermore, the proliferative behaviors of the tumor cells were evaluated by immunohistochemical staining of Ki-67 in the FFPE tissues of the xenograft tumors. The intensity of Ki-67 staining was elevated in tumors from the group overexpressing miR-423-5p compared with the control group (Fig. 6g). However, a notable reduction in MTSS1 expression was also demonstrated in the miR-423-5p overexpression group compared to the control group (Fig. 6g). To continue the examination of the clinical significance of MTSS1 expression, we evaluated MTSS1 expression by immunohistochemical analysis of identical FFPEs of the 68 LAD specimens. MTSS1 exhibited negative or weak staining in LAD tissue with BM. In contrast, MTSS1 displayed positive staining in LAD tissue without BM (Fig. 6h). Moreover, MTSS1 expression was positively correlated with the lymph node metastasis ratio of LAD (Table 5). We also conducted Spearman correlation coefficient analysis to determine the association of MTSS1 expression level and miR-423-5p expression in LAD tissue samples. The expression levels of miR-423-5p showed an inverse correlation with the upregulated expression levels of MTSS1 in LAD specimens (Fig. 6i). Additionally, Kaplan–Meier survival analysis revealed an association between low MTSS1 expression and poor BM survival in patients with LAD (Fig. 6j).

**Table 5:** Correlation between MTSS1 expression and clinicopathological parameters of LAD patients with and without BM

Characteristics	MTSS1 expression status		
	Low (*n* = 38)	High (*n* = 30)	*P*
Gender			
Male	25 (65.8%)	17 (56.7%)	0.442
Female	13 (34.2%)	13 (43.3%)	
Age			
≤60	15 (39.5%)	14 (46.7%)	0.552
>60	23 (60.5%)	16 (53.3%)	
Tumor stage			
T1 + T2	35 (92.1%)	27 (90.0%)	0.761
T3	3 (7.9%)	3 (10.0%)	
Histologic grade			
Well/moderate	26 (68.4%)	20 (66.7%)	0.878
Poor/NS	12 (31.6%)	10 (33.3%)	
Lymph node ratio			
≤1/3	19 (44.2%)	24 (76.0%)	0.011
>1/3	19 (55.8%)	6 (24.0%)	
Brain metastasis			
BM	25 (58.1%)	5 (20.0%)	0.002
NBM	18 (41.9%)	20 (80.0%)	

*BM* brain metastasis, *NBM* non-brain metastasis, *NS* not stated

## Discussion

The process of the metastatic cascade is complex and involves multiple steps^18^. Metastatic diseases involving the central nervous system have become increasingly more treatable. As better control over systemic disease occurs, brain metastases have become more widespread, and large molecules such as antibodies are used to control primary cancers^19^. Because of the blood-brain barrier (BBB) and the brain’s distinctive microenvironment, new treatment strategies for BM are required. MiRNAs are a large group of small non-coding RNAs 19–25 nucleotides in length that are generated after larger strands of RNA are cut. MiRNAs attach to complementary sequences in the 3‵UTR of genes and induce the formation of protein complexes that are involved in preventing translation and/or lowering mRNA stability^20^. In cancer cells, miRNAs have unusual expression patterns compared with healthy cells. MiRNA profiles can discriminate between primary and secondary brain tumors and classify the original metastatic brain tumor^21^. These discoveries indicate that miRNA profiling can be utilized to determine cancer cell origin and their reaction to therapy.

In this study, we observed that miR-423-5p was upregulated in LAD samples with BM compared with primary LAD without BM. Then we investigated the relationship between miR-423-5p and clinicopathological features and survival in patients with NSCLC. The clinical parameters; namely, gender, T stage, histologic grade and lymph node ratio, did not show a significant correlation with BM. Kaplan–Meier analysis revealed that the high miR-423-5p expression was linked to poor BM survival in LAD patients. Multivariate Cox proportional hazard regression analysis of each parameter suggested that high miR-423-5p expression resulted in significantly adverse prognostic factors that were independent of BM. The altered expression of a series of NSCLC-associated miRNAs was identified in previous studies (e.g., miR-675-5p, miR-200, and miR-1253)^22–25^. However, miRNAs associated with the risk of developing BM in NSCLC have not been extensively researched. Although some BM-associated miRNAs (e.g., miR-328, miR-145-5p, and miR-378) have been reported in NSCLC using miRNA arrays^17,26,27^, these studies were only performed on a small number of specimens. Therefore, the elevated expression of miR-423-5p in LAD samples with BM suggests that this miRNA may play a role in “brain-seeking” metastatic potential^28,29^, which may be involved in the development of BM from primary LAD. Therefore, miR-423-5p shows potential as a biomarker.

To better understand the underlying role of miR-423-5p in LAD, we characterized the biological function of miR-423-5p in vitro and in vivo. In this study, we showed that the overexpression of miR-423-5p promoted the colony formation, motility, migration, and invasion capacity of LAD cells. In contrast, the downregulation of miR-423-5p had the opposite effects. Moreover, the overexpression of miR-423-5p induced tumorigenesis and BM in nude mice. These results show that miR-423-5p is indispensable in the growth and metastasis of LAD. Metastasis-related deaths account for approximately 90% of cancer mortalities^30^. Accumulating evidence has shown that miRNAs participate in tumor growth and/or metastasis, and a growing number of miRNAs have been implicated in lung cancer metastasis^31,32^. The role of miRNAs in the biology of BM has been documented in prior evaluations of numerous primary tumor types^33–37^. In lung cancer, miRNA-145 downregulation participated in the progression of LAD and induced the development of BM^38^. MiRNA-378 promoted BM in NSCLC by increasing the expression levels of matrix metalloproteinase 7 (*MMP-7)*, *MMP-9*, and vascular endothelial growth factor (*VEGF)* and by decreasing the levels of suppressor of fused homolog, all of which are key genes that are involved in angiogenesis and invasion of the extracellular matrix^27^. In NSCLC, miRNA-328 modulates cell migration and the development of BM via altered expression of the PKC alpha genes^17^. Comparably, in breast cancer, the migratory and invasive ability of breast CSCs is linked to Krueppel-like factor 4 expression, which is inversely related to the expression of miRNA-7^39^. MiR-1258 changes are directly linked to the expression of heparanase, which is an established prometastatic enzyme found in BM in breast cancer cells that degrades chains of heparan sulfate to alter the cytoskeleton and makes it easier for cells to cross the BBB^40,41^.

The mechanism by which miRNAs change gene expression is still debatable, but many studies have indicated that miRNAs are mainly processed by the RNA-mediated interference machinery to initialize target gene mRNA degradation, in full or in part^42,43^. Previous mechanistic studies of miRNAs in NSCLC with BM have primarily focused on the expression status of epidermal growth factor receptor, K-RAS, VEGF, and MMP-9^44–47^. Current bioinformatics analysis demonstrated that miR-423-5p might attach to the 3’UTR of MTSS1, and we noted that MTSS1 expression might be repressed by miR-423-5p. MTSS1, which has been mapped to chromosome 8q24.1 in the human genome, encodes a 5.3-kb mRNA and a polypeptide that is believed to be a 356 amino acid actin-binding protein that is homologous to members of the Wiscott-Aldrich Syndrome protein family^48,49^. MTSS1 functions as a tumor suppressor in certain human cancers, including lung, bladder, prostate, and gastric cancers^50–54^. Recently, the functions of MTSS1 have been investigated in prostate cancer cell lines and have been shown to significantly reduce cell migration and proliferation^55^. Comparably, MTSS1 is a possible mechanistic target of miR-23a since increased MTSS1 expression is tightly linked with metastatic disease in tumors from colorectal cancer patients^56,57^. The combination of miR-423-5p functional analysis with MTSS1 in animal models will assist in the additional investigation of their roles in metastasis and show the clinical treatment value for LAD patients; this will be the aim of our future research studies. Evaluations of human samples have increased the pertinence of miR-423-5p modulation on MTSS1 in LAD by showing the inverse correlation of the expression of these molecules. The ability of MTSS1 overexpression to counteract the pro-invasion effects of miR-423-5p clearly indicates the importance of their relationship in LAD metastasis. MTSS1 levels were lower in lung cancer tumors with BM than in those without BM. Low MTSS1 levels correlated with the lymph node metastasis ratio. In addition, low MTSS1 expression levels in lung cancer tumors were associated with reduced patient BM survival.

In conclusion, this is the first report to show an association between miR-423-5p and BM in LAD and to show that miR-423-5p is a potentially useful BM biomarker. Because patients expressing miR-423-5p might be at highest risk for BM, these patients are most likely to benefit from PCI. Furthermore, the inhibitor knock down of miR-423-5p could be therapeutically beneficial for increasing the expression of MTSS1 in LAD.

## Methods

### Clinical tissue samples

The FFPE specimens of 155 cases containing 87 clinical samples from LAD patients (training group) and 68 validation LAD samples (test group) were obtained from the Cancer Hospital, Chinese Academy of Medical Sciences (Beijing, China) between 2003 and 2008. Each of the patients underwent surgical resection and adjuvant therapy based on the standard of care. Of them, 62 patients experienced later brain metastasis, and 93 patients did not have BM. Stages were documented based on the 6^th^ edition American Joint Committee on Cancer staging system. Two pathologists independently assessed the tumors’ histologic type, grade, and percentage using hematoxylin and eosin (H&E)-stained specimens. Patients’ clinical characteristics are shown in Table 1. Written informed consent was acquired from patients so their biological materials could be utilized. This evaluation was authorized by the Institutional Review Board of the Cancer Institute and Hospital.

### MicroRNA microarray assay

The analysis of miRNA microarray data was conducted in 32 clinical samples acquired from LAD patients with BM in contrast to 55 patients without BM. In short, total RNA isolated from patient samples was examined with the mammalian miRNA array V2.0 (CapitalBio, Beijing, China), which identified 1105 miRNAs in humans, mice, and rats. Separation of low-molecular-weight RNAs from total RNA was conducted by the polyethylene glycol precipitation method; then low-molecular-weight RNAs were labeled with 5-phosphate-cytidyl-uridyl-Cy3-3 and hybridized to the mammalian miRNA array overnight at 42 °C. The LuxScan 10 K/A laser confocal scanner was used to scan the arrays, and the acquired images were evaluated using LuxScan 3.0 software (both from CapitalBio). Cluster 3.0 was used to conduct the clustering analysis, and the results were viewed with TreeView software.

### Cell culture and transfection

Five LAD cell lines (A549, NCI-H1299, NCI-H157, ANIP-973, and PC-9) were obtained from the Cell Culture Center of Peking Union Medical College (Beijing, China). Human embryonic kidney (HEK) 293 T cells were purchased from ATCC (Manassas, VA, USA). LAD cell lines were cultured in RPMI-1640 medium, and HEK 293 T cells were maintained in DMEM supplemented with 10% fetal bovine serum (Gibco BRL, Grand Island, NY) in a humidified atmosphere of 5% CO_2_ at 37 °C.

### RNA extraction and qPCR analyses

Total RNA was extracted from cell lines and frozen tumor specimens using TRIzol (Invitrogen, CA, USA) according to the manufacturer’s instructions. The qPCR primers were devised as revealed in Supplementary Table S1. qPCR assays were conducted to identify miR-423-5p and MTSS1 expression using the QuantiTect SYBR Green PCR Kit (Takara, Tokyo, Japan) and a StepOne Real-Time PCR System (Applied Biosystems, Foster City, CA, USA) based on the manufacturer’s instructions. The relative levels of miR-423-5p and MTSS1 were determined by qPCR utilizing gene-specific primers. U6 or GAPDH was included as a control for normalization. MiR-423-5p and MTSS1 levels were respectively normalized to U6 and GAPDH, to produce a 2-⊿⊿CT or 2-⊿CT value for each transcript (Bio-Rad CFX manager software 3.1). Experiments were conducted a minimum of three times.

### Western blotting

Total protein was extracted by cell lysis in RIPA buffer with added protease inhibitor. Separation of protein samples was carried out by sodium dodecyl sulfate polyacrylamide gel electrophoresis, and the proteins were transferred to polyvinylidene fluoride membranes. The membranes were probed with anti-MTSS1 or anti-GAPDH (ab78161, ab9485; Abcam, Cambridge, UK) after blocking in 5% bovine serum albumin and then incubated with horseradish peroxidase (HRP)-conjugated secondary antibody (goat-anti-mouse IgG [1:2000] and goat-anti-rabbit IgG [1:3000]). Proteins were detected with the Image Reader LAS-4000 (Fujifilm) and examined with Multi Gauge V3.2 software.

### MiRNA transfection

Every endogenous mature miRNA mimic and inhibitor was bought from RiboBio (Guangzhou, China). Lipofectamine 2000 (Invitrogen) was used in the transient transfections of miRNA mimics/inhibitors using the company’s instructions. At 6 h after transfection, the culture media was substituted for transfection media. Each of the miRNA transfections was incubated for 48 h.

### Colony formation and wound healing assays

MiR-423-5p mimic or mimic negative control (NC) and miR-423-5p inhibitor or inhibitor NC were used to transfect LAD cells. After 24 h, the transfected cells were trypsinized, quantified, and replated at a density of 1 × 10^3^ cells/10 cm plate. After 10 days, developing colonies were fixed in 3.7% methanol and stained with 0.1% crystal violet for quantification. Colonies with a minimum of 50 cells were scored. For the wound healing assay, at 24 h following the transfection into LAD cells with miR-423-5p mimics, inhibitors, and controls, the cells were seeded in identical amounts onto 6-well culture plates in RPMI 1640 medium along with 1% fetal bovine serum (FBS) at about 95% confluence. At 12 h after seeding, a vertical wound was produced with a 10 µL pipette tip. Images were obtained at specific times (0, 18, and 42 h) to determine the gap closure rate. Each assay was conducted three times.

### Cell migration and invasion assay

For the invasion assays, LAD cells that had been transfected with miR-423-5p mimics, inhibitors, and controls for 24 h were collected in medium along with 1% FBS, after which they were plated in BD BioCoat BD Matrigel Invasion Chambers (BD China, Shanghai, China) at 5 × 10^4^ cells per chamber. Matrigel (BD China) covered the membrane in the chamber. Medium with added 10% FBS was placed in the bottom chamber. After 24 h of incubation, the cells that did not enter the membrane’s pores were removed with a cotton swab. Cells on the membrane’s bottom surface were fixed with polyoxymethylene (Sigma) and stained with 0.1% crystal violet (Sigma) for 0.5 h. Quantification of the stained cells was performed under a microscope in eight randomly chosen fields, and the average was utilized to determine whether cell invasion had occurred. Identical inserts without Matrigel were utilized to examine cell invasive potential in the invasion assay.

### Construction of plasmids

The pDonR223-MTSS1 plasmid bearing the human MTSS1 gene was bought from Changsha Axybio Bio-Tech Co., Ltd. (Changsha, China). The pDonR223-MTSS1 plasmid was used for amplification of the full coding sequence of human MTSS1. The MTSS1 amplification products and the pEGFP-N1 plasmid were digested with XhoI and Hind III, and T4 DNA ligase was used to purify and ligate the DNA fragments. Once ligated, the product was transformed into TOP10 competent cells; the positive clone was labeled pEGFP-N1-MTSS1.

### Generation of stable cell lines

Recombinant lentiviral vectors with knock down of miRNA-423-5p and irrelevant sequences were acquired from Hanbio Biotechnology (Shanghai, China). Luciferase and puromycin reporter genes driven by the EF1α promoter were used to confirm timely infection efficiency. To establish lentiviral vectors, the precursor sequence for miRNA-423-5p and an irrelevant sequence (NC) were inserted into the pHBLV-U6-MCS-EF1α-Luc-T2A-puromycin lentiviral vectors. Recombinant lentiviruses were packaged by co-transfection of HEK-293T cells with pSPAX2 and pMD2.G with LipoFilter^TM^ reagent. The supernatant with lentivirus particles was collected 48 and 72 h after transfection and passed through 0.45-μm cellulose acetate filters (Millipore, Stafford, VA, USA). Ultracentrifugation of recombinant lentiviruses was used for their concentration. To prepare stable cell lines, LAD cells were transduced with lentivirus with an MOI of about 50 in 5 μg/mL polybrene. The supernatant was removed after 24 h and new complete culture medium was substituted. Infection efficiency was verified by qPCR at 96 h after infection, and the cells were chosen with 2 μg/mL puromycin for 2 weeks.

### Luciferase reporter assay

LAD cells were transiently transfected with the expression plasmids of reporter vectors with MTSS1 3‵-UTR or their mutants, as well as miR-423-5p or control mimics, using Lipofectamine 2000 (Invitrogen) according to the manufacturer’s instructions. At 48 h post-transfection, 100 µL Passive Lysis Buffer (Promega, Fitchburg, WI, USA) was used to lyse the cells, and 20 µL lysate was used to assay luciferase utilizing the dual luciferase reporter assay on the Berthold FB12 luminometer (Berthold, Bad Wildbad, Germany) as previously described. Normalization of alterations in Renilla luciferase expression was conducted by measuring firefly luciferase activities.

### Bioinformatics methods and mRNA expression profiling

We performed mRNA microarray experiments to investigate the differential expression of mRNA profiles after transfection into LAD cells with miR-423-5p mimics and controls. Snap-frozen cells were shipped to CapitalBio (CapitalBio) for processing and analysis with use of Affymetrix 3’IVT. Data processing and determination of differentially expressed genes were performed essentially as described.

### LAD mouse model

The animal studies were performed out using 5-week-old BALB/C-nude mice, which were kept in specific pathogen-free conditions. For the in vivo tumor proliferation assay, 1 × 10^6^ H157 cells that had been transfected with Lenti-miR-423-5p or Lenti-NC were injected subcutaneously into the nude mice (five mice per group). The growth of tumors was monitored via measurement with calipers 1–2 times a week for at least 4 weeks. Tumor volume was calculated as V = L × l^2^ × 0.5, where L and l represent larger and the smaller tumor diameters, respectively. For the in vivo experimental metastasis model, transfected cancer cells (1 × 10^6^ in 100 μL HBSS) were injected into the tail vein directly. After 6 weeks, tumor colony formation in the subcutaneous tissues was noted by H&E staining and histology examination. To evaluate the development and metastasis of the implanted tumor cells, bioluminescence images were acquired. The in vivo bioluminescence signal was quantified by anesthetizing mice with isoflurane prior to in vivo imaging, and D-luciferin solution (150 mg/kg in PBS, In Vivo Imaging Solution; PerkinElmer, Waltham, MA, USA) was intravenously injected for subcutaneous and systemic xenografts. The IVIS Spectrum imaging system (PerkinElmer) was used to acquire the bioluminescence images 2–5 min following the injection, and the images were quantified by measuring the photon flux (photons/s/cm^2^/steradian) for a region of interest around the bioluminescence signal using the Living Image Software package (Perkin Elmer/Caliper Life Sciences).

### Immunohistochemical staining

Immunohistochemistry (IHC) was conducted on 4-μm thick slices using the EnVision two-step technique of the Dako REAL™ Envision™ Detection System (Dako, Agilent Technologies, Santa Clara, CA, USA). The slides were treated with the following solutions: primary antibodies, namely, Ki-67 (1:50, ab15580; Abcam, Cambridge, UK) and MTSS1 (1:50), HRP-labeled secondary antibody and diaminobenzidine. Protein staining was examined at 400× magnification with a light microscope. Two knowledgeable pathologists manually scored the staining intensity as follows: 0, absence of staining; 1, weak staining; 2, moderate staining; and 3, strong staining. Tumor cells from five fields were chosen randomly, and the percentage of positively stained cells (0–100%) was determined. The ultimate IHC score was determined by multiplying the intensity score by the percentage of positive cells.

### Statistical analysis

All of the values documented in the study are expressed as the means ± standard deviation (SD), and the error bars depict the SD of the mean. Student’s *t*-test, *χ*^2^ test, and repeated measures analysis of variance were utilized to establish significance. The log-rank test was utilized to examine the impact of the clinical variables and miRNAs on BM-free survival. The Cox regression model was utilized to examine the impact of the related factors on the survival time of NSCLC patients. A *P* value < 0.05 was considered statistically significant. Statistical analyses were conducted with SPSS 16.0 software (SPSS Inc., Chicago, IL, USA).

## Electronic supplementary material

Supplementary Table 1

## References

[1] Jemal A (2011). Global cancer statistics. CA.

[2] Anglim PP, Alonzo TA, Laird-Offringa IA (2008). DNA-methylation-based biomarkers for early detection of non-small cell lung cancer: an update. Mol. Cancer.

[3] Ding X (2012). Risk factors of brain metastases in completely resected pathological stage IIIA-N2 non-small cell lung cancer. Radiat. Oncol..

[4] Schoutten LJ, Rutten J, Huveneers HA, Twijnstra A (2002). Incidence of brain metastasis in a cohort of patients with carcinoma of breast, colon, kidney, and lung and melanoma. Cancer.

[5] Mujoomdar A (2007). Clinical predictors of metastatic disease to the brain from non-small cell lung carcinoma: primary tumor size, cell type, and lymph node metastases. Radiology.

[6] Sperduto PW (2012). Summary report on the graded prognostic assessment: an accurate and facile diagnosis specific tool to estimate survival for patients with brain metastases. J. Clin. Oncol..

[7] Laack NN, Brown PD (2004). Cognitive sequelae of brain radiation in adults. Semin Oncol..

[8] Oh Y (2009). Number of metastatic sites is a strong predictor of survival in patients with nonsmall cell lung cancer with or without brain metastases. Cancer.

[9] Knights EM (1954). Metastatic tumors of the brain and their relation to primary and secondary pulmonary cancer. Cancer.

[10] Nugent JL (1979). CNS metastases in small cell bronchogenic carcinoma: Increasing frequency and changing pattern with lengthening survival. Cancer.

[11] Pugh TJ, Gaspar LE (2007). Prophylactic cranial irradiation for patients with lung cancer. Clin. Lung Cancer.

[12] Gore EM (2011). Phase III comparison of prophylactic cranial irradiation versus observation in patients withlocally advanced non-small-cell lung cancer: primary analysis of radiation therapy oncology group study RTOG 0214. J. Clin. Oncol..

[13] Ambros V (2004). The functions of animal microRNAs. Nature.

[14] Zanetti KA (2012). 3’-UTR and functional secretorhaplotypesinmannose- bindinglectin2areassociatedwith increased colon cancer risk in African Americans. Cancer Res.

[15] Tominaga N (2015). Brain metastatic cancer cells release microRNA-181c-containing extracellular vesicles capable of destructing blood-brain barrier. Nat. Commun..

[16] Zhang H, Li Y, Lai M (2010). The microRNA network and tumor metastasis. Oncogene.

[17] Arora S (2011). MicroRNA-328 is associated with (non-small) cell lung cancer (NSCLC) brain metastasis and mediates NSCLC migration. Int. J. Cancer.

[18] Chaffer CL, Weinberg RA (2011). A perspective on cancer cell metastasis. Science.

[19] Barnholtz-Sloan JS (2004). Incidence proportions of brain metastases in patients diagnosed (1973 to 2001) in the metropolitan detroit cancer surveillance system. J. Clin. Oncol..

[20] Lewis BP, Burge CB, Bartel DP (2005). Conserved seed pairing, often flanked by adenosines, indicates that thousands of human genes are microrna targets. Cell.

[21] McDermott R, Gabikian P, Sarvaiya P, Ulasov I, Lesniak MS (2013). Micrornas in brain metastases: big things come in small packages. J. Mol. Med (Berl.).

[22] He D (2015). Down-regulation of miR-675-5p contributes to tumor progression and development by targeting pro-tumorigenic GPR55 in non-small cell lung cancer. Mol. Cancer.

[23] Kim JS, Kurie JM, Ahn YH (2015). BMP4 depletion by miR-200 inhibits tumorigenesis and metastasis of lung adenocarcinoma cells. Mol. Cancer.

[24] Gasparini P (2015). microRNA classifiers are powerful diagnostic/ prognostic tools in ALK, EGFR, and KRAS-driven lung cancers. Proc. Natl. Acad. Sci. USA.

[25] Remon J (2016). miRNA-197 and miRNA-184 are associated with brain metastasis in EGFR-mutant lung cancer. Clin. Transl. Oncol..

[26] Donzelli S (2015). Epigenetic silencing of miR-145-5p contributes to brain metastasis. Oncotarget.

[27] Chen LT (2012). MicroRNA-378 is associated with non-small cell lung cancer brain metastasis by promoting cell migration, invasion and tumor angiogenesis. Med. Oncol..

[28] Langley RR, Fidler IJ (2011). The seed and soil hypothesis revisited—the role of tumor-stroma interactions in metastasis to different organs. Int J. Cancer.

[29] Lorger M (2012). Tumor microenvironment in the brain. Cancers.

[30] Mehlen P, Puisieux A (2006). Metastasis: a question of life or death. Nat. Rev. Cancer.

[31] Leidinger. P, Keller A, Meese E (2012). MicroRNAs-Important Molecules in Lung Cancer Research. Front Genet..

[32] Hayes J, Peruzzi PP, Lawler S (2014). MicroRNAs in cancer: biomarkers, functions and therapy. Trends Mol. Med.

[33] Zhang X (2011). MicroRNA-19 (miR-19) regulates tissue factor expression in breast cancer cells. J. Biol. Chem..

[34] Creighton CJ (2010). Molecular profiling uncovers a p53-associated role for microRNA-31 in inhibiting the proliferation of serous ovarian carcinomas and other cancers. Cancer Res.

[35] Gregory PA (2008). The miR-200 family and miR-205 regulate epithelial to mesenchymal transition by targeting ZEB1 and SIP1. Nat. Cell Biol..

[36] Mott JL, Kobayashi S, Bronk SF, Gores GJ (2007). Mir-29 regulates Mcl-1 protein expression and apoptosis. Oncogene.

[37] Li L (2014). Hypoxia-induced miR-210 in epithelial ovarian cancer enhances cancer cell viability via promoting proliferation and inhibiting apoptosis. Int. J. Onco.

[38] Zhao C (2013). Down-regulation of miR-145 contributes to lung adenocarcinoma cell growth to form brain metastases. Oncol. Rep..

[39] Okuda H (2013). miR-7 suppresses brain metastasis of breast cancer stem-like cells by modulating KLF4. Cancer Res.

[40] Ridgway LD, Wetzel MD, Ngo JA, Erdreich-Epstein A, Marchetti D (2012). Heparanase-induced GEF-H1 signaling regulates the cytoskeletal dynamics of brain metastatic breast cancer cells. Mol. Cancer Res..

[41] Zhang L, Sullivan PS, Goodma JC, Gunaratne PH, Marchetti D (2011). MicroRNA-1258 suppresses breast cancer brain metastasis by targeting heparanase. Cancer Res..

[42] Tsujiura M (2010). Circulating microRNAs in plasma of patients with gastric cancers. Br. J. Cancer.

[43] Hausser. J, Zavolan M (2014). Identification and consequences of miRNA-target interactions [mdash] beyond repression of gene expression. Nat. Rev. Genet.

[44] Benedettini E (2010). Met activation in non- small cell lung cancer is associated with de novo resistance to EGFRinhibitors and the development of brain metastasis. Am. J. Pathol..

[45] Cassoni P (2009). Caveolin- 1 expression in lung carcinoma varies according to tumour histotype and is acquiredde novo in brain metastases. Histopathology.

[46] Munfus-McCray D (2011). EGFR and KRAS mutations in metastatic lung adenocarcinomas. Hum. Pathol..

[47] Fidler IJ, Yano S, Zhang RD, Fujimaki T, Bucana CD (2002). The seed and soil hypothesis: vascularisation and brain metastases. Lancet Oncol..

[48] Wang J (2012). MicroRNA-182 downregulates metastasis suppressor 1 and contributes to metastasis of hepatocellular carcinoma. BMC Cancer.

[49] Lin J (2005). Differential regulation of cortactin and N-WASP-mediated actin polymerization by missing in metastasis (MIM) protein. Oncogene.

[50] Kayser G (2015). Downregulation of MTSS1 expression is an independent prognosticator in squamous cell carcinoma of the lung. Br. J. Cancer.

[51] Du P, Ye L, Ruge F, Yang Y, Jiang WG (2011). Metastasis suppressor-1, MTSS1, acts as a putative tumour suppressor in human bladder cancer. Anticancer Res.

[52] Nixdorf S (2004). Expression and regulation of MIM (missing in metastasis), a novel putative metastasis suppressor gene, and MIM-B, in bladder cancer cell lines. Cancer Lett..

[53] Loberg RD (2005). Differential expression analysis of MIM (MTSS1) splice variants and a functional role of MIM in prostate cancer cell biology. Int J. Oncol..

[54] Liu K (2010). Downregulation of metastasis suppressor 1(MTSS1) is associated with nodal metastasis and poor outcome in Chinese patients with gastric cancer. BMC Cancer.

[55] Mustafa N, Martin TA, Jiang WG (2011). Metastasis tumour suppressor-1 and the aggressiveness of prostate cancer cells. Exp. Ther. Med..

[56] Jahid S (2012). miR-23a promotes the transition from indolent to invasive colorectal cancer. Cancer Discov..

[57] Wang D (2011). MTSS1 overexpression correlates with poor prognosis in colorectal cancer. J. Gastrointest. Surg..

