# Supplementary material for: MiR-423-5p in brain metastasis: potential role in diagnostics and molecular biology
Source: Cell Death Dis. 2018 Sep 17;9(10):936. doi: 10.1038/s41419-018-0955-5 (PMC6141540; doi:10.1038/s41419-018-0955-5)
Supplement: Supplementary file 1 — Supplementary Table 1 [file 41419_2018_955_MOESM1_ESM.doc]

TableS1. Specific primers are as follows:

| ANTXR1 | F: ACTCCAGGTCAGCATGAACG |
| --- | --- |
|  | R:GCAGGCGGTATTCTCTGCTA |
| BANK1 | F:GAGAAGGGATGTAAGAGCAGGC |
|  | R:GTACAGAGCCCATTCCTCAGC |
| CASP1 | F:CAGGGGAACAGTGGTTCACA |
|  | R:TCAGGGTAGGAGGGGAATGG |
| CCNG2 | F:GGTGAGGCTACAGTGATTCCA |
|  | R:CAAGGCACAGATGCCAAACC |
| CEACAM1 | F:GACACTGGAATCTCCATCCGTT |
|  | R:GAGTGGTCCTGAGTGTGGTT |
| DUSP5 | F: CCAGCTTATGACCAGGGTGG |
|  | R:CGAGGAACTCGCACTTGGAT |
| EGF | F:AGAGCTTGGAGGACAACAGC |
|  | R:GCAGGACCCACACAAGTAGA |
| FAS | F:CACCACCATCCTGAACAAAGG |
|  | R:GGTGATGCCATTACGCTCCT |
| GAB1 | F: CTGCCATTAACTGTGCTTCCC |
|  | R:GCTGGCTGGAGGAGTAACAG |
| IGFBP3 | F:GCGCCAGGAAATGCTAGTGA |
|  | R:GGGGTGGAACTTGGGATCAG |
| IRF1 | F:GCCATTCACACAGGCCGATA |
|  | R:GTTGTAGCTTCAGAGGTGGAGG |
| ITGB3 | F:ACCAGTAACCTGCGGATTGG |
|  | R:TCCGTGACACACTCTGCTTC |
| JAK2 | F: GGATGTGAGTGGGAGCTGAG |
|  | R: GAAACCGGCTACACAGATCG |
| MAP2 | F:GCACACTCACATCCACCTGA |
|  | R:TCAGCTGCTAAAGGCAGAGC |
| MAP3K8 | F:ACCGGGCAGTCTCTTTCTGTT |
|  | R: GAAGATTGCATCTGCGGCCT |
| MDM2 | F:CGAGCTTGGCTGCTTCTGG |
|  | R:GTACGCACTAATCCGGGGAG |
| MTSS1 | F: ATCCAGCCTCAGTTGGACAG |
|  | R:TCGGCCACGTTCTTCAATCA |
| PDGFRL | F:TGAAGCGGGGCTTTGTGTAT |
|  | R:TGTCTTGGATCGTCACAGGC |
| RASSF10 | F: GTCGTCCTGTTCGTCCACTT |
|  | R:TGTCCTGCACGTAGTTGACC |
| RET | F:CACGAGAGCTGATGGCACTA |
|  | R:TTCACAAAGAAAGGGCCGGT |
| SAMD9 | F:TGGGTGAATAGGTGGCCTTT |
|  | R:TGGTCCATGTGTGATGCCC |
| STAT1 | F:CAGACCACAGACAACCTGCT |
|  | R:TACAGAGCCCACTATCCGAGA |
| TGFB2 | F:TTGTGCTCCAGACAGTCCCA |
|  | R:GCTCAATCCGTTGTTCAGGC |
| TNFAIP3 | F:GCCTACAACCCGCATACAAC |
|  | R:GCGATCCTTTCGCAAAGTCC |
| TNFAIP6 | F:AGAAGCACGGTCTGGCAAAT |
|  | R:GCTGCCTCTAGCTGCTTGTA |
| TNFSF10 | F:TTGGGACCCCAATGACGAAG |
|  | R:TGGTCCCAGTTATGTGAGCTG |
| TP53INP1 | F:CTGTGCATAACTCCTGCCCT |
|  | R:TCTGTGCCCGTGAGTCTTAT |
| TWIST1 | F:TACGCCTTCTCGGTCTGGAG |
|  | R:CCACGCCCTGTTTCTTTGAA |
| WISP2 | F:GCAAGCTGTCACAGGCTCTT |
|  | R:CCAGCTTTGAGCCTGAAGGT |
| XAF1 | F:CAGCCTCAGGAATCAAGGGG |
|  | R: TTCCTGCACACCGAGAAGTC |
